# Nutrition practice, compliance to guidelines and postnatal growth in moderately premature babies: the NUTRIQUAL French survey

**DOI:** 10.1186/s12887-015-0426-4

**Published:** 2015-09-03

**Authors:** Silvia Iacobelli, Marianne Viaud, Alexandre Lapillonne, Pierre-Yves Robillard, Jean-Bernard Gouyon, Francesco Bonsante, for the NUTRIQUAL group

**Affiliations:** Centre d’Etudes Périnatales de l’Océan Indien, CHU La Réunion - Saint Pierre, BP 350 97448 Saint Pierre Cedex, France; Néonatologie, Réanimation Néonatale et Pédiatrique, CHU La Réunion - Saint Pierre, Saint Pierre Cedex, BP 350 97448 France; Department of Neonatology, APHP Necker Enfants Malades Hospital, Paris, France; Paris Descartes University, Paris, France

**Keywords:** Newborn, Survey, Feeding, Calories, Standards, Growth retard, Parenteral and enteral intake

## Abstract

**Background:**

The nutritional care provided to moderately premature babies is poorly studied. For a large cohort of such babies, we aimed to describe: nutrition practice intentions, comparison of the intended with the actual practice, compliance of actual practice to current nutrition guidelines, and postnatal growth.

**Methods:**

A questionnaire was sent out to 29 neonatal intensive care units in France, in order to address practice intentions. In the same units, retrospective patient’s data were collected to assess actual practice, compliance to nutrition guidelines and infant postnatal growth. The cumulative nutritional deficit during the two first weeks of life was calculated and variables associated with ΔZ-score for weight at 36 weeks postconceptional age/discharge (ΔZ-score_w_ 36PCA/DC) were analysed by multivariate linear regression.

**Results:**

276 infants born 30 to 33 weeks of gestation were studied. Among them, 76 % received parenteral nutrition on central venous line after birth. On day of life 1 (DOL1), 93 % of infants had parenteral amino acids (AA), at an intake ≥ 1.5 g/kg in 27 % of cases. Lipids were started at ≤ DOL2 in 47 % of infants. There was a divergence between the intended and the actual practice for both AA and lipids intake. The AA and energy cumulative deficit (DOL1 to DOL14) were respectively 10.9 ± 8.3 g/kg and 483 ± 181 kcal/kg. Weight Z-score (mean ± SD) significantly decreased from birth (−0.17 ± 0.88) to 36 weeks PCA/DC (−1.00 ± 0.82) (p < 0.0001), and the extra-uterine growth retardation (EUGR) rate at 36 weeks PCA/DC was 24.2 %. Independent variables associated with ΔZ-score_w_ 36PCA/DC were AA cumulative intake and DOL of full enteral feeding.

**Conclusions:**

Nutrition intake was not in compliance with recommendations, and the rate of EUGR was considerable in this cohort. Efforts are needed to improve adherence to nutrition guidelines and growth outcome of moderately preterm infants.

## Background

In the last decades, several studies demonstrated serious cumulative nutritional deficit and extra-uterine growth retardation (EUGR) in preterm infants during the first weeks of life [1–3].

More recently, the causes of this growth failure have been explored and in part identified. The implementation of continuous education, the carrying out of surveys to focus on trends in clinical practice [4, 5] and the adherence of postnatal nutrition to current recommendations, have been addressed as possible solutions for improving growth and reducing EUGR [6, 7].

All this literature relates to extremely or very low birth weight (VLBW) infants and very little is known about the nutritional care of the more “healthy” but still immature babies, born between 30 and 33 weeks of gestational age (GA), even though they account for 15 % of preterm life births and 30 % of neonatal intensive care unit (NICU) admissions.

Some reports have demonstrated that these infants, too, fail to achieve intrauterine growth rates during hospital stay and that significant variations in feeding practices and growth outcomes exist among NICUs [8, 9]. However, to date, no study has evaluated the adherence to guidelines for energy and amino acids (AA) intake in this specific population.

Infants within this cohort are generally less “sick” when compared with their very preterm counterparts and they have more subtle feeding issues and complications. Moreover, they often require a limited technological support and the choice of avoiding the central venous line (CVL) insertion – in order to reduce the possible associated complications - may limit the supply of the recommended protein and caloric intake, especially during the transitional period [10].

So, the determinants of postnatal growth and the factors influencing the adequacy of nutrition support in moderately preterm babies may be different from those of VLBW infants and deserve a better understanding.

The aims of this study were the following: 1) to describe nutrition practice intentions in a large cohort of moderately premature babies; 2) to compare nutrition practice intentions to actual practice; 3) to describe the adherence of units’ actual practice to current nutrition guidelines; 4) to measure the growth outcomes during hospital stay in this cohort.

## Methods

### Setting and participants

A survey was carried out in 2014 in French mainland and overseas departments and territories. It was designed to include a heterogeneous group of preterm infants born between 30 and 33 weeks of gestation and cared for in tertiary NICUs or secondary (IIB) level nurseries. For the purpose of this study, infants born between 30 to 33 weeks of gestation were defined “moderately premature babies". The survey consisted of one-page questionnaire and an electronic file to collect patients’ data. The aims of the questionnaire and of the electronic file were respectively to address the unit nutrition practice intentions and to describe the actual adherence to current nutrition guidelines.

The questionnaire was addressed to the senior physician of each unit or to a delegated colleague having clinical experience of neonatal intensive care, and it consisted of multiple choice and open-ended questions. Specifically for infants born at 30–31 and at 32–33 weeks of gestation, the questionnaire assessed the following variables: whether written nutrition guidelines were available in the unit, whether parenteral nutrition (PN) was considered since the day of birth, the type of venous access chosen in case of PN, postnatal day on which parenteral AA and lipid intake was started, AA starting dose, postnatal day on which enteral feeding was started, enteral feeding advancement and fortification strategy.

PN was defined as “intravenous nutrition given through a central or peripheral line and containing at least both dextrose and nitrogen”, full enteral feeding as “enteral feed given as sole nutritional source”. Day of life (DOL) 1 was defined as the day of birth.

In each centre, respondents were asked to record data from the last consecutive 12 patients on the electronic file (3 patients for each GA, born from 30 to 33 weeks of gestation) being at 36 weeks postconceptional age (PCA) or discharged from the unit.

Exclusion criteria were: major or digestive congenital anomalies, outborn, transfer to other hospital within DOL14 and death within the first week of life.

### Data collection and management

The identity of the respondent to the questionnaire and that of the centre’s electronic file were both blinded for the analysis to all authors. Patients’ records were anonymous.

Data recording for this study was approved by the National Committee for data protection (Commission Nationale de l’Informatique et des Libertés, registration number 1687438).

This study was approved by the institutional medical research ethics committee (Comité de Protection des Personnes Sud-Ouest et Outre Mer III, authorization number 2013/76). According to French legislation, written parental consent was not needed for this study.

Demographic features and in-hospital morbidities were collected. The following data were recorded on DOL1, 3, 7, 14 and at 36 weeks PCA or at discharge (DC) (when before 36 weeks PCA): weight, parenteral (g/Kg/d of dextrose, AA and lipids) and enteral intake (ml/kg/d of human or given formula milk), whether a central or peripheral venous line was inserted.

Mean human milk content was assumed to be 64 kcal⁄dl, 1.4 g/dl of protein, 3.2 g/dl of fat and 7.0 g⁄dl of carbohydrate. Human milk fortifier and preterm infant formula compositions were based on the product’s labelled nutritional content.

Head circumference and length were measured at birth and at 36 weeks PCA/DC. Finally, information was retrieved on total days of PN on central and peripheral venous line, DOL on which trophic or enteral feeding was started, DOL on which milk fortification was started, type and amount of milk fortifier, DOL of full enteral feeding and maximal postnatal weight loss.

### Variables of interest and statistical analysis

#### Nutrition practice intentions

Nutrition practice intentions were compared to the actual practice with regard to the following variables: type of venous access at birth, DOL of initiation of enteral feeding, DOL of initiation for parenteral AA and lipids and AA starting dose.

#### Adherence of unit actual practice to current nutrition guidelines

Compliance to nutrition guidelines was measured according to current European recommendations [11] with the theoretical values recently indicated per GA group [12]. Nutrition intake was considered compliant to guidelines if: i) AA initiation dose (≥1.5 g/kg/d) was started at DOL1; ii) AA target dose (≥3.5 g/k/d) was attained by DOL7; iii) energy target dose (≥120 kcal/kg/d) was attained by DOL7.

The cumulative AA (g/kg) and energy intake (Kcal/kg) (DOL1 to DOL14) was estimated according to the formula:

$$ \mathrm{Cumulative}\ \mathrm{intake} = \left(\mathrm{DOL}{1}_{\mathrm{intake}}*1.5\right) + \left(\mathrm{DOL}{3}_{\mathrm{intake}}*3\right) + \left(\mathrm{DOL}{7}_{\mathrm{intake}}\ *5.5\right) + \left(\mathrm{DOL}{14}_{\mathrm{intake}}\ *4\right) $$

#### Formula elaboration and validation

The formula was elaborated based on the assumption that a linear progression of intake occurred between DOL1 to DOL3, DOL3 to DOL7, and DOL7 to DOL14, and it was validated in a subgroup of the cohort study: in 48 infants (from 4 units) the AA and energy intake was collected daily from DOL1 to DOL14 and the actual cumulative intake (DOL1 to DOL14) was correlated to the cumulative intake estimated by the formula by a linear regression procedure. The model showed a very good regression coefficient (*r*^2^ = 0.9729; *p* < 0.001 and 0.9648; *p* < 0.001 respectively for AA and energy intake).

In all infants, the cumulative deficit (DOL1 to DOL14) for AA and energy was calculated as following:

$$ \mathrm{Cumulative}\ \mathrm{deficit} = \left(\mathrm{Target}\ \mathrm{dose}*14\right) - \mathrm{Cumulative}\ \mathrm{intake} $$

For the purpose of some analyses, infants and centres were split into tertiles of respectively AA cumulative intake and mean AA cumulative intake.

#### Growth outcomes

Z-score [mean and standard deviation (SD) for GA] for weight, length and head circumference was calculated at birth and at 36 weeks PCA/DC, according to the reference [13].

Infants were considered SGA when the sex- and age-adjusted weight at birth was below the 10^th^ percentile according to the reference [13].

They were defined EUGR when they were not born SGA and the sex- and age-adjusted weight at birth and at 36 weeks PCA/DC was below the 10^th^ percentile according to the reference [13].

Simple and multiple linear regressions were performed to investigate variables associated with the delta Z-score for weight at 36 weeks PCA/DC (ΔZ-score_w_ 36PCA/DC).

Potential cofounders were selected among antenatal (steroids administration, preeclampsia, diabetes, labour, mode of delivery), clinical (GA, gender, singleton birth, SGA, surfactant administration, phototherapy, hypoglycemia, Apgar ≤ 3 at 1 min, neonatal morbidities) and nutritional (CVL insertion at birth, DOL of enteral feeding initiation, DOL of full enteral feeding, tertile of AA cumulative intake, center tertile for mean AA cumulative intake, cumulative energy intake) factors. These were included in the multivariate model only if they were significant at a *p* value < 0.10. The coefficient of determination (r^2^) of the model was calculated, in order to choose the best-fit equation for the data set.

Comparisons between groups were performed using *χ*^2^-test (or Fisher’s exact test) for categorical variables; the ANOVA test was used for parametric variables and the Mann–Whitney *U* test for non-parametric continuous variables.

All statistical analyses were carried out using the MedCalc. ver. 12.3.0.0 statistical software package (MedCalc Software Mariakerke, Belgium) and *p* values <0.05 were considered statistically significant.

## Results

### Questionnaire responses and study population

A total of 29 units were surveyed. Four units were secondary excluded as they only provided responses to the questionnaire without feedback of patients’ data.

So, finally, 25 units were included in the analysis (23 III level NICUs and 2 IIB level nurseries).

Data of 276 patients were analysed. Population characteristics are presented in Table 1.

**Table 1:** Population characteristics (*n* = 276 preterm infants born 30–33 weeks gestation)

Antenatal variables	
Preeclampsia, n (%)	68 (25.5)
Maternal Diabetes, n (%)	35 (12.8)
Antenatal Steroids, n (%)	251 (91.6)
Singleton Pregnancy, n (%)	62 (22.2)
Infant characteristics	
Birth Weight (g), mean ± SD	1582 ± 375
Length (cm), mean ± SD	39.9 ± 3.1
Head Circumference (cm), mean ± SD	28.4 ± 3.9
Gestational Age, n (30/31/32/33)	64/68/76/68
Small for gestational age, n (%)	33 (12)
Male Gender, n (%)	135 (48.9)
Caesarean Section, n (%)	175 (63.6)
Apgar score ≤ 3 at 5 min, n (%)	6 (4.0)
Hypoglycemia requiring treatment, n (%)	36 (13.3)
Phototherapy, n (%)	219 (79.6)
Surfactant Administration, n (%)	85 (30.8)
Mechanical Ventilation, n (%)	91 (33.3)
Early Onset Sepsis, n (%)	12 (4.4)
Late Onset Sepsis, n (%)	29 (10.5)
Necrotizing enterocolitis, n (%)	4 (1.4)
Intraventricular haemorrhage grade 1–2, n (%)	35 (12)
Intraventricular haemorrhage grade 3–4, n (%)	2 (0.7)
Cystic periventricular leukomalacia, n (%)	5 (1.8)
Hospital Stay (days), mean ± SD	32.5 ± 15.8

Table 2 shows the questionnaires responses (intended practice) according to groups of GA.

**Table 2:** Questionnaires responses (intended practice) according to groups of GA (25 units)

	30-31 weeks	32-33 weeks
Written unit guidelines, n (%)	18 (72)	19 (76)
PN started on DOL1, n (%)	22 (88)	14 (56)
CVL for PN, n (%)	11 (44)	4 (16)
AA parenteral initiation, DOL (mean ± SD)	1.08 ± 0.4	1.13 ± 0.6
AA parenteral initiation dose, g (mean ± SD)	1.39 ± 0.59	1.31 ± 0.58
Lipid parenteral initiation, DOL (mean ± SD)	2.0 ± 0.6	2.1 ± 0.4
Enteral Feeding Initiation, DOL (mean ± SD)	1.3 ± 0.5	1.2 ± 0.4
Enteral Feeding Advancement, ml/kg/d (mean ± SD)	18.2 ± 5.0	19.6 ± 5.6
Mother milk fortification, n (%)	23 (92)	19 (76)

### Nutritional actual practice

Among the 276 infants, 92 % had a PN and 76 % a CVL for PN. The mean duration of PN on CVL was 9.0 ± 8.1 days.

The average time of initiation of enteral feeding was 1.7 ± 1.2 DOL. Feeding volume (mean ± SD) was advanced from 9 ± 12 ml/kg/d on DOL1 to 38 ± 26 ml/kg/d on DOL3, reaching 87 ± 48 ml/kg/d at DOL7 and 140 ± 50 ml/kg/d by DOL14. The average day of full enteral feeding was 11.7 ± 6.9 DOL. A total of 73 % of all study subjects received human milk during their hospital stay and 42 % were at least partially breastfed at 36 weeks PCA/DC. The average time of initiation of milk fortifier was 9.9 ± 5.5 DOL. The global rate of milk fortification was 65 % and 36 % of all infants received fortified feeding at 36 weeks PCA/DC.

Parenteral AA were started on DOL1 in 93 % of infants, the administered dose at DOL1 was ≥ 1.5 g/kg in 27 % and lipid administration was started at ≤ DOL2 in 47 %. The results on the AA and lipid administration practice and the comparison of the intended and actual practice according to groups of GA are presented in Table 3.

**Table 3:** Comparison of the intended and actual practice according to groups of GA (276 infants)

	30-31 weeks		32-33 weeks	
	Intended Practice	Actual Practice	Intended Practice	Actual Practice
CVL for PN (%)	63	92	31	61
Enteral feeding initiation DOL (mean)	1.3	1.9	1.2	1.5
PN started on DOL1 (%)	88	90	56	73
AA parenteral initiation at DOL1 (%)	88	94	88	92
AA parenteral initiation dose ≥ 1.5 g/kg/d (%)	41	33	35	22
Lipid parenteral initiation ≤ DOL2 (%)	91	54	82	41

For the all population study, the AA intake (g/kg/d) was 1.1 ± 0.7 at DOL1; 2.9 ± 0.9 at DOL7, and the energy intake (kcal/kg/d) 94 ± 18 at DOL7. The cumulative AA (g/kg) and energy intake (kcal/kg) were respectively 37.0 ± 8.3 and 1201 ± 166 and the correspondent cumulative deficits were 10.9 ± 8.3 and 483 ± 181.

### Growth outcomes

Table 4 details study population Z-scores and ΔZ-scores for anthropometric measures at birth and at 36 weeks PCA/DC.

**Table 4:** Z-scores and ΔZ-scores in 276 preterm infants born 30–33 weeks of gestation

	Birth Z-score	Z-score 36 weeks PCA/DC	ΔZ-score^a^	p^b^
Weight (mean ± SD)	−0.17 ± 0.88	−1.00 ± 0.82	−0.80 ± 0.47	<0.0001
Length (mean ± SD)	−0.21 ± 1.14	−1.12 ± 1.09	−0.90 ± 0.91	<0.0001
Head Circumference (mean ± SD)	0.04 ± 1.04	−0.41 ± 0.92	−0.41 ± 0.74	<0.0001

^a^ΔZ-score from birth to 36 weeks PCA/DC. ^b^Z-score 36 weeks PCA/DC versus Birth Z-score (ANOVA for repeated measures)

For the all population study, the maximal postnatal weight loss was 8.3 ± 3.9 %. At 36 weeks PCA/DC the rate of babies whose weight was below the 10^th^ percentile according to the reference^13^ was 36.7 % (12.5 % of infants being born SGA and 24.2 % having EUGR).

Variables associated with ΔZ-score_w_ 36PCA/DC at the univariate analysis were: infant tertile for AA cumulative intake, centre tertile for mean AA cumulative intake, cumulative caloric intake, DOL of full enteral feeding, SGA, GA, labour, Apgar ≤ 3 at 1 min of life, phototherapy (data not shown). Variables associated with ΔZ-score at 36 weeks PCA/DC at the multivariate analysis are detailed in Table 5.

**Table 5:** Variables associated with ΔZ-score_w_ at 36 weeks PCA/DC in 276 preterm infants born 30–33 weeks of gestation. Multiple linear regression, general coefficient of the model *r* ^2^ = 0.10, *p* < 0.001

Variables	Coefficient	r ^partial^	p
Tertile for AA cumulative intake	0.13	0.22	<0.001
DOL of full enteral feeding	−0.01	−0.18	0.005
Small for Gestational Age	0.16	0.12	0.06
Gestational Age	0.05	0.12	0.07

Variables associated with cumulative deficit of protein intake (DOL 1–14) at the multivariate analysis are detailed in Table 6.

**Table 6:** Variables associated with cumulative deficit of protein intake (DOL 1–14) at the multivariate analysis in 276 preterm infants born 30–33 weeks of gestation

Variables	Coefficient	r ^partial^	p
Center tertile for mean AA intake	−4.3	0.50	<0.001
PN in CVL at day1	−3.8	0.27	<0.001
DOL of full enteral feeding	−0.23	0.23	<0.001
Gestational Age	1.3	−0.22	<0.001
Small for Gestational Age	−2.8	0.14	0.01
Preeclampsia	−1.8	0.13	0.03

## Discussion

This study shows the poor postnatal growth of a moderately premature population, demonstrating that both EUGR rate and cumulative nutritional deficit are considerable during the first weeks of life in infants born 30 to 33 weeks of gestation. The main result is that a better nutritional intake may significantly reduce the ΔZ-score_w_ from birth to 36 weeks PCA/DC in this vulnerable population.

Critical illness, insufficient early PN support, lack of education and training in PN prescription have been proven responsible for the growth retardation acquired during hospitalization of extremely and VLBW infants [1, 14–16].

To our knowledge, this is the first time that the impact of all these factors on the growth outcome has been explored for the specific population of more healthy but still immature preterm babies. Indeed, several clinical and nutritional variables potentially influencing the degree of growth failure have been investigated by this report.

Studies in extremely low birth weight infants have underlined that insufficient early PN support and EUGR are a serious problem, especially for neonates who are small, immature and critically ill, and that they are independently associated to acute morbidities and illness conditions (i.e. need for assisted ventilation, necrotizing enterocolitis) [1].

Recently, Santerre and Rigo [6, 7] have shown that early postnatal nutrition is critical to limit the EUGR and that nutritional policy and growth can be optimized by the respect of current recommendations, even in babies with severe prematurity-related morbidities.

Their observation was assessed in a cohort of extremely and VLBW infants, but based on our results, it may also apply to a more healthy population of larger babies and more advanced GA.

Our study has also documented that shorter time to achieve full enteral feeding was significantly associated with a more favourable growth outcome. This is consistent with the results of Ehrenkranz et al. [17], in a prospective cohort of VLBW infants and those of Blackwell et al. [8], in a large cohort of healthy, premature infants born between 30 and 35 weeks of gestational age. In this retrospective study, which did not take into account the use of PN, earlier initiation of enteral feeding and higher feeding volumes over the first week of life were correlated with lower weight loss during that period.

Interesting also, in our study, cumulative protein deficit appears significantly lower in babies with lower gestational ages and with intrauterine growth retardation, it is inversely associated with centre tertile for mean AA intake and with the use of CVL on DOL1, and it is not influenced by prematurity-related morbidities.

According to all the above data, differences in growth in our population would more clearly reflect differences in nutritional care and may reveal more effective feeding strategies among centres. This result has interesting implications for clinicians and NICU policies.

In an original way, this study aimed to evaluate the adherence of the intended and the actual practices to the recommended PN guidelines for preterm infants, even if the information was collected within the limits of the starting administration of AA and lipids. It is worthwhile to note that, whether both the practices for DOL of starting AA were in compliance with the established PN guidelines, this was not the case for the AA starting dose, or for lipids administration. This is an important point to be addressed in the course of continuous education strategies, because, as noticed in a recent report from Fischer and coll. [18], early parenteral lipid intake is positively associated with weight gain in extremely low birth weight infants and may improve early nutritional support of preterm neonates.

Finally, our results somehow show a discrepancy between what physicians intend to prescribe and what they do really provide to preterm babies, and this leads to the following considerations: the first one regards the methodological limitation of using surveys for the assessment of nutritional protocols, as already highlighted by previous reports [4, 15, 19]; the second one concerns the need of greater efforts to implement the existing known guidelines at the local level in NICUs.

The present study has several limitations. The major one is undoubtedly that our data may not be considered as representative of the entire population of the moderately premature babies targeted by the survey, especially with regards to results of intention-to-treat practices, as questionnaires were not distributed on a national basis, contrary to most surveys [8, 15]. A further point is that some information were not available in our report, that is the rate of daily nutriment progression, the unit contraindications to enteral and parenteral feeding advancement, the presence of metabolic abnormalities (acidosis, hyperglycemia, hypertriglyceridemia) during the first days of life, the incidence of sepsis associated to CVL and finally the type of solutions, additive and supplements used in PN. The model of multiple linear regression used to identify factors associated with ΔZ-score_w_ at 36 weeks PCA/DC has a quite poor general coefficient, suggesting that other variables not considered in the analysis may account for explaining the growth outcome in the studied population. Finally, the present investigation was not designed to explore enteral feeding practices in detail in terms of fortification strategies, feeding techniques and breastfeeding polices, which might produce differences in growth outcome, as illustrated by other studies [20, 21].

## Conclusions

The results of our report have interesting implications for clinicians and NICU policymakers. As already demonstrated for extremely and VLBW infants, our study allows to conclude that the nutrition delivered to infants born 30–33 weeks gestation is often not in compliance with international guidelines, and that this is associated with considerable rates of EUGR.

The reasons for suboptimal AA and total calories delivery in this specific population mainly reside on unit nutrition practice and feeding strategies, this making that the growth outcome may be improved.

Factors influencing EUGR in moderately premature babies are not fully elucidated, and further studies are needed to identify practices associated with better growth in this quite healthy, but still vulnerable infant population.

## Abbreviations

AA: Amino acids
CVL: Central venous line
DC: Discharge
DOL: Day of life
ΔZ-score_w_ 36PCA/DC: ΔZ-score for weight at 36 weeks postconceptional age/discharge
EUGR: Extra-uterine growth retardation
GA: Gestational age
NICU: Neonatal intensive care unit
SD: Standard deviation
SGA: Small for gestational age
VLBW: Very low birth weight
PCA: Postconceptional age
